# Predictors of long-acting injectable antipsychotic treatment discontinuation in outpatients with schizophrenia: relevance of the Drug Attitude Inventory-10

**DOI:** 10.1097/YIC.0000000000000359

**Published:** 2021-04-23

**Authors:** Lorenzo Tatini, Giulio D’Anna, Francesco Pietrini, Eugenia Calligaris, Andrea Ballerini, Valdo Ricca

**Affiliations:** a Psychiatry Unit, Department of Health Sciences, University of Florence; b Department of Mental Health and Addictions, Central Tuscany NHS Trust, Florence, Italy

**Keywords:** DAI-10, long-acting injectable antipsychotics, patient-reported outcome measures, schizophrenia, treatment discontinuation

## Abstract

Given the importance of patients’ subjective experience and attitudes in the management of severe mental illness, the present study evaluated their potential role as predictors of future continuation of long-acting injectable antipsychotic maintenance treatment (LAI-AMT) in clinically stable outpatients with schizophrenia switching from an oral therapy. Retrospective data from 59 subjects receiving LAI-AMT for at least 6 months were collected. Patients who continued LAI treatment (*n* = 32) were compared to those who discontinued it (*n* = 27), assessing baseline socio-demographic and clinical characteristics, psychopathological features (Positive And Negative Syndrome Scale, Montgomery–Åsberg Depression Rating Scale and Young Mania Rating Scale) and patient-reported experience of treatment through Drug Attitude Inventory 10-item (DAI-10) and Subjective Well-being under Neuroleptics short form. Binary logistic and Cox regression analyses explored the predictive role of the mentioned variables on treatment discontinuation. The Kaplan–Meier estimator compared dropout from LAI treatment in subsamples with different characteristics. Unemployment and lower baseline DAI-10 scores predicted LAI-AMT discontinuation. No major differences were detected in other socio-demographic, clinical or psychometric indexes. When switching from oral to LAI-AMT, the preliminary assessment of attitude towards drug might be clinically relevant, allowing the identification of patients at risk for treatment discontinuation.

## Introduction

Long-acting injectable (LAI) antipsychotics play a key role in the maintenance treatment of schizophrenia (Brissos *et al*., 2014), which is frequently influenced by reduced compliance (Kane *et al*., 2013). In fact, LAIs proved to reduce relapses and hospitalizations, mostly by addressing suboptimal adherence to pharmacological therapies (Correll *et al*., 2016; Tiihonen *et al*., 2017). For this reason, some experts suggest that LAI antipsychotics should be considered and systematically proposed to any patients for whom maintenance antipsychotic treatment is indicated (Llorca *et al*., 2013), and recent clinical guidelines underline that their use is not limited to poor compliance to oral therapies (Galletly *et al*., 2016; Barnes *et al*., 2020; Gaebel *et al*., 2020).

Conversely, LAI utilization in clinical practice must consider several factors, which may counterbalance their advantages over oral therapy. For instance, some patients are sceptical about LAIs (Jaeger and Rossler, 2010), and discontinuation rates remain considerable (Rittmannsberger *et al*., 2017). Moreover, LAI formulations per se are more expensive than their oral counterpart, even though their long-term pharmaco-economic impact might result in a global reduction of costs (Kaplan *et al*., 2013). Because this decreased burden is less likely to be appreciated in the short term, early treatment discontinuation should be avoided.

Given these relevant drawbacks, identifying early predictors of treatment persistence in LAI antipsychotic maintenance therapy (AMT) appears to be a major goal in clinical practice. Previous studies outlined that illness-related factors such as severe psychopathology are commonly implicated in treatment discontinuation (Yang *et al*., 2012; Brain *et al*., 2013), and anamnestic data (e.g. recent psychiatric admissions before LAI treatment) influence the subsequent therapy continuity (Berardi *et al*., 2019). Socio-demographic factors also play a role, as seen for family and social support (Rabinovitch *et al*., 2009), and for employment (Verdoux *et al*, 2000; Brain *et al*., 2013).

In this framework, patients’ attitudes and perspectives are often overlooked, and their practical value may be underestimated. For instance, an early improvement of subjective well-being is a major predictor of compliance to AMT (Naber, 2008), and a positive attitude toward drugs represents a predictor of adherence in multi-episode (Yang *et al*., 2012; Brain *et al*., 2013) and first-episode patients (Gaebel *et al*., 2010). The relevance of the abovementioned factors has mostly been investigated in oral AMT. For instance, the 30-item form of the Drug Attitude Inventory (DAI; Hogan *et al*., 1983) allowed clinicians to identify patients at risk of treatment discontinuation (Gaebel *et al*., 2010; Yang *et al*, 2012), whereas the 10-item version score (DAI-10; Nielsen *et al*., 2012), together with good psychosocial functioning, already proved to predict future medication adherence (Brain *et al*., 2013).

When initiating LAI-AMT, baseline DAI scores may identify patients at risk of treatment discontinuation. Because DAI-10 scores showed significant changes over time in patients who were switched from oral to LAI-AMT (Pietrini *et al*., 2018), an early acquisition of this information may prove to be clinically useful.

The aim of the present study was to carry a retrospective analysis on clinically stable outpatients with schizophrenia who were switched from oral to LAI-AMT, to identify potential baseline differences – in socio-demographic, clinical and psychometric characteristics – which may influence future compliance, with a focus on self-reported experience of treatment.

## Material and methods

The present study retrospectively collected data from clinically stable outpatients with schizophrenia who were about to be switched to LAI-AMT, on the basis of their clinical management (Llorca *et al*., 2013). All patients attending the LAI Outpatient Facility of Florence University Hospital for this clinical purpose were 18–65 years old at the time of switching. Patients were included in the retrospective analysis if they met the following criteria:

(1) Diagnosis of schizophrenia (Diagnostic and Statistical Manual of mental disorders, Fifth edition; American Psychiatric Association, 2013)
(2) Patients first entered the facility between January 2013 and January 2017 (to ensure that their course of illness after LAI initiation could be evaluated for at least 3 years)
(3) Patients had been stabilized on a single antipsychotic treatment with oral olanzapine, risperidone, paliperidone or aripiprazole for at least 4 weeks before switching to LAI-AMT
(4) Patients had received at least 6 months of LAI treatment with a stable posology, to ensure an adequate pharmacological trial (in terms of pharmacokinetic steady state, clinical efficacy and tolerability)
(5) Patients had been enrolled in the ‘LAI antipsychotics on Functioning and Experience’ (LAI-FE) study to evaluate baseline patient-reported outcomes (PROs); further details on the LAI-FE study inclusion and exclusion criteria are provided elsewhere (Pietrini *et al*., 2018).

The evaluation of potential predictors of treatment persistence included baseline socio-demographic data (age, gender, marital status, employment and years of instruction) and clinical history (duration of illness, number of past hospitalizations, number of previous antipsychotic treatments, current antidepressant or mood stabilizing treatment). Before switching, patients’ psychopathology was evaluated through the Positive and Negative Syndrome Scale (PANSS; Kay *et al*., 1987), and affective symptoms were assessed through the Montgomery–Åsberg Depression Rating Scale (MADRS; Montgomery and Åsberg, 1979) and the Young Mania Rating Scale (YMRS; Young *et al*., 1978). These scales contributed to the definition of clinical stability utilized as the inclusion criterion in the LAI-FE study (Pietrini *et al*., 2018):

(1) Outpatient status
(2) PANSS total score ≤ 120 (Leucht *et al*., 2005)
(3) MADRS total score < 30 (Müller *et al*., 2003)
(4) YMRS total score < 25 (Lukasiewicz *et al*., 2013)
(5) A score ≤ 4 on each of the following PANSS items: delusions (P1), conceptual disorganization (P2), suspiciousness (P3), hallucinatory behaviour (P6) and unusual thought content (G9)
(6) A score ≤2 on item 10 of the MADRS (‘Weary of life. Only fleeting suicidal thoughts’).

Moreover, the baseline scores of the DAI-10 (Nielsen *et al*., 2012) and the Subjective Well-being under Neuroleptics short form (SWN-K; Naber *et al*., 2001) were used to evaluate patients’ attitudes and subjective experience of treatment immediately before switching from oral to LAI-AMT.

Before the collection of data, participants provided written informed consent. Patients’ anonymity was always ensured. The study protocol was approved by the ethics committee of the local institution, and it was conducted in accordance with the current International Conference on Harmonization of Technical Requirements for Good Clinical Practice guidelines, as contained in the Declaration of Helsinki.

Of the 78 patients with schizophrenia who were switched to LAI-AMT between January 2013 and January 2017, 19 patients were excluded from the analysis because their baseline PROs were not available. Therefore, 59 patients were included in the analysis, and the duration of treatment – in months – was recorded. Each patient attended regular psychiatric evaluations and drug administration. Discontinuation of LAI treatment was defined as reaching an interval of 40 days because the previous LAI-AMT administration, due to patients’ decision.

The final sample was divided into two subgroups: the ‘continued treatment’ subgroup (*n* = 32) included patients who did not interrupt their LAI treatment when analyses were conducted (May, 2020), whereas the ‘discontinuation of treatment’ subgroup (*n* = 27) included patients who interrupted their LAI treatment at any time.

### Statistical analyses

Continuous variables were reported as mean ± SD (M ± SD). Categorical variables were reported as absolute and relative frequency.

To assess between-group differences, Student’s *t*-test and Pearson’s chi square (*χ*^2^) test were performed where appropriate.

To evaluate the potential role of the analysed factors in predicting treatment continuation, those variables for which the *P* value of the *t*-test or chi square test was <0.20 were included in a binary logistic regression analysis with treatment discontinuation (‘Yes’ or ‘No’) as the dichotomous variable. This arbitrary cutoff was chosen to produce an inclusive model, and to account for the small sample size which resulted in a low statistical power. A Cox regression analysis was carried for the same variables tested in the binary logistic regression, with follow-up time as the dependent variable, and treatment discontinuation as the recorded event.

To conclude, a Kaplan–Meier survival plot examined those variables for which the regression analyses led to the rejection of the null hypothesis. For this analysis, the total sample (*N* = 59) was divided into two groups on the basis of the median score for continuous variables, whereas no further splitting was required for dichotomous variables. Survival plots with dropout from treatment as the recorded event were traced, accompanied by a log-rank test.

For each analysis, the null hypothesis was rejected at an alpha value <0.05. All statistical analyses were performed using the Statistical Package for Social Sciences (SPSS) version 25 (IBM Corp., 2017).

## Results

The analysed sample consisted of 59 patients. At the time of switching, 35 of them were treated with olanzapine (59.3%), 3 with risperidone (5.1%), 18 with paliperidone (30.5%) and 3 with aripiprazole (5.1%). Patients were switched to the corresponding dose of LAI-AMT: olanzapine pamoate for olanzapine, paliperidone palmitate for risperidone or paliperidone and aripiprazole monohydrate LAI for aripiprazole. The global characteristics of the sample are presented in Table 1. The mean follow-up time in months was 45.61 ± 30.65 for the total sample. The difference between the ‘continued treatment’ subgroup (*n* = 32; 62.91 ± 21.45 months) and the ‘discontinuation of treatment subgroup’ (*n* = 27; 25.56 ± 27.13 months) was significant (*t* = 5.91; *P* < 0.001).

**Table 1:** Patients’ characteristics at the time of switching from oral to long-acting injectable antipsychotic maintenance treatment, and differences between the ‘continued treatment’ and the ‘discontinuation of treatment’ subgroups

	Total sample (*N* = 59)	Continued treatment (*n* = 32)	Discontinuation of treatment (*n* = 27)	*t* or *χ*^2^	*P* value
Socio-demographic data					
Age (years)	37.61 ± 12.21	38.75 ± 10.10	36.26 ± 14.40	0.76	0.454
Male gender	32 (54.2%)	18 (56.3%)	14 (52.9%)	0.11	0.735
Single	39 (66.1%)	23 (72.9%)	16 (59.3%)	1.04	0.308
Employed^a^	29 (49.2%)	20 (62.5%)	9 (33.3%)	**4.98**	**0.026**
Instruction (years)^a^	12.44 ± 3.39	12.97 ± 3.36	11.81 ± 3.38	1.31	0.195
Clinical history					
Duration of illness (years)	15.98 ± 11.28	17.41 ± 10.32	14.30 ± 12.30	1.06	0.295
Number of past hospitalizations^a^	2.36 ± 1.61	2.06 ± 1.37	2.70 ± 1.82	−1.55	0.128
Number of previous antipsychotic trials	2.58 ± 1.43	2.41 ± 1.39	2.78 ± 1.48	−1.00	0.324
Antidepressant	30 (50.8%)	11 (34.4%)	9 (33.3%)	0.01	0.933
Mood stabilizer	25 (42.4%)	15 (46.9%)	10 (37.0%)	0.58	0.446
Psychopathology					
PANSS total score	61.49 ± 23.57	57.84 ± 24.29	65.81 ± 22.36	−1.30	0.198
p-PANSS^a^	13.95 ± 6.35	13.41 ± 6.50	14.59 ± 6.22	−0.71	0.479
n-PANSS^a^	14.29 ± 8.14	12.69 ± 7.84	16.19 ± 8.22	−1.67	0.100
g-PANSS^a^	33.02 ± 11.94	31.56 ± 12.58	34.74 ± 11.12	−1.02	0.313
MADRS	14.90 ± 9.20	14.56 ± 9.63	15.30 ± 8.84	−0.30	0.763
YMRS	6.15 ± 7.72	6.25 ± 6.15	6.04 ± 9.38	0.11	0.917
Patient-reported-outcomes					
DAI-10^a^	1.71 ± 5.58	3.81 ± 4.72	−0.78 ± 5.58	**3.43**	**0.001**
SWN-K total score	75.95 ± 20.55	73.66 ± 23.36	78.67 ± 16.66	−0.93	0.355
SWN-K emotional regulation^a^	15.76 ± 4.80	14.88 ± 5.30	16.81 ± 3.97	−1.57	0.123
SWN-K self-control^a^	15.46 ± 4.44	14.59 ± 4.75	16.48 ± 3.88	−1.65	0.104
SWN-K mental functioning^a^	15.39 ± 4.83	15.03 ± 5.15	15.81 ± 4.48	−0.62	0.540
SWN-K physical functioning^a^	14.86 ± 4.46	15.09 ± 4.73	14.59 ± 4.19	0.43	0.671
SWN-K social integration^a^	14.73 ± 4.46	14.53 ± 5.03	14.97 ± 3.76	−0.37	0.714

DAI-10, Drug Attitude Inventory 10-item version; g-PANSS, general subscale of the PANSS; MADRS, Montgomery–Åsberg Depression Rating Scale. n-PANSS, negative subscale of the PANSS; PANSS, Positive And Negative Syndrome Scale; p-PANSS: positive subscale of the PANSS; SWN-K, Subjective Well-being under Neuroleptics, short form; YMRS, Young Mania Rating Scale.

a Variables included in the subsequent regression analyses.

Bold indicates statistically significant results.

### Between-group comparison

Differences between clinical subgroups are reported in Table 1. Age at initiation of LAI treatment, gender and marital status did not differ, whereas the employment rate was significantly lower among subjects who interrupted their LAI treatment (*χ*^2^ = 4.98; *P* = 0.026). The inconclusive difference in years of instruction (*t* = 1.31, *P* = 0.195) was further evaluated in regression analyses.

Clinical history at the time of enrolment did not outline major differences in any of the variables presented, except for a tendency towards a higher number of previous hospitalizations in patients who discontinued LAI treatment (*t* = −1.55; *P* = 0.128), which was included in subsequent regression analyses.

The clinician-administered psychometric scales did not outline major baseline differences. However, the PANSS total score (*t* = −1.30; *P* = 0.198) and the negative symptoms subscale (n-PANSS; *t* = −1.67; *P* = 0.100) showed a tendency towards a different distribution between groups. For this reason, the PANSS subscales were included in regression analyses. No differences were observed in the MADRS and the YMRS scores.

To conclude, the DAI-10 score at baseline proved to be significantly different between subgroups (*t* = 3.43; *P* = 0.001), whereas the SWN-K total score did not outline major differences. However, because two of the subscales presented minor variations, the five SWN-K subscales were included in regression analyses.

### Binary logistic regression

A binary logistic regression with discontinuation of treatment as dichotomic dependent variable (‘Yes’ or ‘No’) was carried for variables with comparisons outlining a *P* value <0.20 in the between-group analysis, including the psychometric indexes for which any of the total or subscale scores met this criterion (Table 2). Dropout from treatment was associated with a higher number of past hospitalizations and with higher scores in emotional regulation. Conversely, a protective effect was observed for employment and higher DAI-10 scores. None of the other variables outlined a significant effect.

**Table 2:** Odds ratio for long-acting injectable treatment discontinuation, based on a binary logistic regression with dropout from treatment (‘Yes’ or ‘No’) as dichotomous dependent variable

	Wald	*P* value	OR (95% CI)
Employed	**4.72**	**0.030**	**0.15 (0.03–0.83**)
Instruction (years)	3.38	0.066	0.81 (0.65–1.02)
Number of past hospitalizations	**5.44**	**0.020**	**2.41 (1.15–5.06**)
p-PANSS	1.69	0.194	0.86 (0.68–1.08)
n-PANSS	0.89	0.345	1.08 (0.92–1.28)
g-PANSS	0.04	0.840	1.01 (0.90–1.14)
DAI-10	**8.52**	**0.004**	**0.71 (0.57–0.89**)
SWN-K emotional regulation	**3.99**	**0.046**	**1.40 (1.01–1.95**)
SWN-K self-control	1.62	0.203	1.29 (0.87–1.92)
SWN-K mental functioning	0.01	0.956	1.01 (0.75–1.36)
SWN-K physical functioning	2.71	0.099	0.79 (0.60–1.05)
SWN-K social integration	1.85	0.174	0.80 (0.58–1.10)

CI, confidence interval; DAI-10, Drug Attitude Inventory 10-item version; g-PANSS, general subscale of the PANSS; n-PANSS, negative subscale of the PANSS; OR, odds ratio; PANSS, Positive And Negative Syndrome Scale; p-PANSS, positive subscale of the PANSS; SWN-K, Subjective Well-being under Neuroleptics, short form.

Bold indicates statistically significant results.

### Survival analysis

A Cox regression analysis with dropout from treatment as the recorded event during the follow-up explored the contribution of the same covariates tested in *Binary logistic regression* (Table 3). The analysis highlighted a protective role of higher DAI-10 scores and employment against LAI-AMT discontinuation, whereas results were not conclusive for the other variables.

**Table 3:** Hazard ratio for long-acting injectable treatment discontinuation, based on a Cox regression analysis with dropout from treatment as recorded event during follow-up time

	Wald	*P*	Hazard ratio (95% CI)
Employed	**8.46**	**0.004**	**0.21 (0.07–0.60**)
Instruction (years)	0.42	0.518	0.96 (0.85–1.09)
Number of past hospitalizations	0.70	0.403	1.14 (0.84–1.53)
p–PANSS	2.42	0.120	0.93 (0.84–1.02)
n-PANSS	0.18	0.673	0.98 (0.91–1.07)
g-PANSS	1.27	0.260	1.03 (0.98–1.08)
DAI-10	**6.35**	**0.012**	**0.88 (0.80–0.97**)
SWN-K emotional regulation	2.11	0.146	1.12 (0.96–1.31)
SWN-K self-control	0.16	0.687	1.05 (0.82–1.35)
SWN-K mental functioning	0.06	0.807	0.97 (0.79–1.21)
SWN-K physical functioning	1.71	0.192	0.90 (0.78–1.05)
SWN-K social integration	0.01	0.974	1.00 (0.85–1.17)

CI, confidence interval; DAI-10, Drug Attitude Inventory 10-item version; g-PANSS, general subscale of the PANSS; n-PANSS, negative subscale of the PANSS; PANSS, Positive And Negative Syndrome Scale; p-PANSS: positive subscale of the PANSS; SWN-K, Subjective Well-being under Neuroleptics, short form.

Bold indicates statistically significant results.

**Fig. 1:**
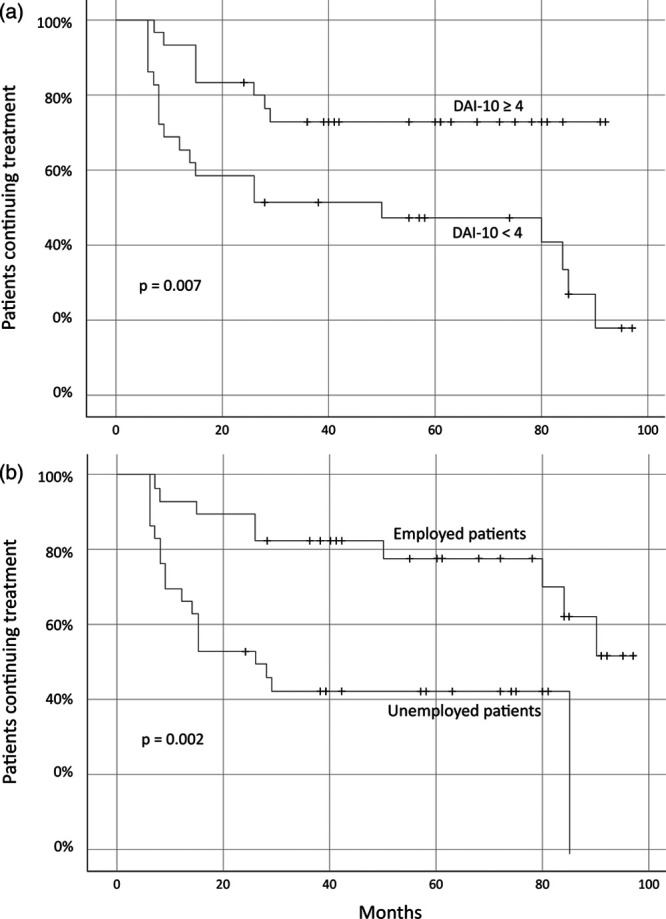
Kaplan–Meier plots describing discontinuation of long-acting injectable treatment among different clinical subgroups; *P* values for the associated log-rank test are reported in each panel. (Panel a) Compares patients who had a baseline Drug Attitude Inventory-10 (DAI-10) score ≥4 with patients who had a baseline DAI-10 score <4. (Panel b) Compares employed patients with unemployed patients.

A Kaplan–Meier estimator plot was traced for the covariates which proved to be statistically significant in any of the regression analyses, and the associated log-rank test was performed (Fig. 1). Because the median DAI-10 score of the sample was 4, patients with a DAI-10 score ≥4 (*n* = 30) were compared to patients with a DAI-10 score <4 (*n* = 29; Fig. 1a), and employed patients (*n* = 29) were compared to unemployed patients (*n* = 30; Fig. 1b). Patients with DAI-10 ≥4 fared significantly better in the survival analysis as compared to patients with DAI-10 <4 (log-rank test: χ^2^ = 7.25; *P* = 0.007; Fig. 1a), and so did employed patients as compared to unemployed patients (log-rank test: χ^2^ = 9.90; *P* = 0.002; Fig. 1b). No differences were detected when the Kaplan–Meier survival analysis and log-rank test compared patients with scores above (*n* = 29) and under (*n* = 30) the median SWN-K emotional regulation subscale score (m >16 versus m ≤16; log-rank test: *χ*^2^ = 0.27; *P* = 0.607), and patients with a higher number of hospitalizations (*n* = 24) versus patients with a lower number of hospitalizations (*n* = 35), as defined by the median value (comparison: m >2 versus m ≤2; log-rank test: *χ*^2^ = 0.97; *P* = 0.324).

## Discussion

AMT discontinuation is a condition of major clinical interest (Kane *et al*., 2013). Even if LAIs avoid covert nonadherence, dropout from this form of treatment remains common, constituting a relevant problem in real-world practice (Olfson *et al*., 2007; Rittmannsberger *et al*., 2017). For this reason, identifying early predictors of treatment persistence is fundamental before initiating this form of AMT. Most guidelines focus on clinical factors when discussing the appropriateness of LAI-AMT prescription (e.g. history of noncompliance, previous relapses and hospitalizations), but patients’ attitudes and perspectives are receiving increasing consideration (Llorca *et al*., 2013). In this light, shared decision-making is crucial in severe mental illness (Deegan and Drake, 2006; Fiorillo *et al*., 2020), potentially shaping attitudes toward antipsychotic medications (Sugawara *et al*., 2019).

Regarding socio-demographic data, the present study showed that being employed predicts treatment persistence. This finding confirms the importance of a multidisciplinary intervention which considers nonpharmacological factors, including social interventions, and it is in line with previous studies outlining the protective role of the occupational status (Verdoux *et al*., 2000; Brain *et al*., 2013). Even if the present study did not evaluate whether unemployment represented a proxy of severity of illness or functional impairment, this anamnestic information should be kept into account whenever possible. Conversely, the present study did not confirm the findings of a previous work with a similar design, where an older age and not being single predicted a longer treatment persistence (Rittmannsberger *et al*., 2017) – most probably due to the small sample size.

In line with an earlier study (Brain *et al*., 2013), the evaluation of clinical history before switching to LAI-AMT did not seem to clearly predict future compliance to treatment. Among the variables presented, only the number of previous hospitalizations proved to play a minor role in the binary logistic regression analysis, with a higher burden among those who discontinued LAI-AMT – in line with a previous study on oral AMT discontinuation (Rittmannsberger *et al*., 2017).

Regarding the psychopathological state of patients at the time of switching, affective symptoms did not differ between groups, as supported by the lack of major differences in concurrent medications. In other words, even if affective symptoms in patients suffering from schizophrenia can be relevant (van Os and Kapur, 2009), they did not prove to have a predictive role in the present observational study. However, the increasing use of LAI for schizoaffective disorder and bipolar disorder demands extreme caution when generalizing these findings to different populations (Sajatovic et al., 2018a, 2018b). PANSS scores showed a variability between the two groups, especially for negative symptoms, which are known to be targeted only marginally by pharmacological treatment, thus, contributing to the burden of functional impairment: these findings may be linked to the abovementioned different employment rates, but they appeared to be less informative *per se*. Moreover, baseline positive symptoms did not stand as predictors of future continuation of LAI-AMT, differently from previous studies on oral AMT (Verdoux *et al*., 2000; Yang *et al*., 2012, Brain *et al*., 2013).

To conclude, the analysis of PROs showed that the baseline SWN-K total score and subscales did not prove to be clearly informative regarding future treatment discontinuation, whereas the mean DAI-10 scores highlighted a remarkable detection property in this sense. Within the sample, using the median value of 4 as cut-off score provided a further, clear characterization of subgroups with different outcomes. For these reasons, the administration of DAI-10 may provide a valuable screening of patients’ attitudes for those subjects whose clinical history and personal preference lead to evaluate LAI-AMT prescription. This informative property of DAI is in line with previous studies on oral AMT (Gaebel *et al*., 2010; Yang *et al*., 2012, Brain *et al*., 2013), and the fact that a longitudinal research outlined the possibility of progressive and sustained improvements of attitude towards drug during LAI-AMT (Pietrini *et al*., 2018) adds importance to the present finding.

Strategies to improve adherence involve personalized support-service interventions, such as electronic reminders, cognitive-behavioural interventions, compliance training and motivational interviews (El-Mallakh and Findlay, 2015). Even if no specific technique was implemented in this observational study, these methods may likely improve attitude towards treatment, and therefore compliance. More in general, the extensive use of adherence-focused sessions already proved to ameliorate compliance in patients with schizophrenia (Barkhof *et al*., 2012), and the monthly administration of LAIs may constitute an opportunity to provide these tailored interventions. Besides, it may be hypothesized that this regular contact with healthcare providers may improve therapeutic alliance, with a positive influence on compliance (Frank and Gunderson, 1990).

To the best of our knowledge, this was the first study to outline a clear role for a self-reported instrument as an early predictor of LAI treatment persistence in clinically stable outpatients with schizophrenia. Because the place of LAIs in clinical practice is strongly influenced by patients’ beliefs, and by psychiatrists’ familiarity with the appropriateness of therapeutic resource (Patel *et al*., 2010; Kirschner *et al*., 2012), DAI-10 may prove useful in the context of a shared decision-making process (Fiorillo *et al*., 2020): as a complement to a thorough anamnestic and clinical evaluation, it may ease the detection of patients at risk for treatment discontinuation, at least for clinically stable subjects which are treated in an outpatient setting.

Regarding significant results, findings on previous hospitalizations and emotional regulation were supported only by the binary logistic regression, but not by survival analyses: this may be linked to the fact that the latter considered the duration of treatment persistence – a main variable of interest – rather than simply assess dropout from LAI-AMT as a dichotomous variable. In this sense, survival analyses represent a more reliable measurement of the outcome of interest, and the fact that they consistently outlined the role of employment and DAI-10, both as continuous and dichotomized scores, confirms the solidity of the findings.

Some limitations of the present study should be addressed. First, this work intentionally avoided head-to-head comparisons of different LAIs, but it is not possible to exclude that part of the phenomena observed might depend on specific drug properties (i.e. different previous oral therapies might have exerted an influence on the baseline evaluation). Second, the small sample size may have led to underpowered analyses, failing to detect factors which played a minor but non-negligible role in determining subsequent adherence to LAI-AMT: this should be kept into account for each nonsignificant result, which should be interpreted as inconclusive, rather than an indication of a lack of differences. Third, 19 subjects were excluded because their PROs were not available, which may be a proxy of severity of illness (e.g. refusal to report subjective experience due to higher levels of suspiciousness, cognitive and functional impairment): this limitation warrants further investigation in studies which does not solely focus on the relevance of PROs.

## Acknowledgements

Conception and design of the study: L.T. and G.D.; acquisition, analysis and interpretation of data: L.T., G.D. and E.C.; drafting the manuscript: L.T., G.D., F.P. and A.B.; manuscript revision and project supervision: F.P., A.B. andV.R.

## Conflicts of interest

There are no conflicts of interest.
